# Distinct Fgf21 Expression Patterns in Various Tissues in Response to Different Dietary Regimens Using a Reporter Mouse Model

**DOI:** 10.3390/nu17071179

**Published:** 2025-03-28

**Authors:** Xinhui Zhang, Zixuan Li, Shuying Wang, Yan Chen

**Affiliations:** CAS Key Laboratory of Nutrition, Metabolism and Food Safety, Shanghai Institute of Nutrition and Health, University of Chinese Academy of Sciences, Chinese Academy of Sciences, Shanghai 200031, China; zhangxinhui2020@sinh.ac.cn (X.Z.); lizixuan2019@sibs.ac.cn (Z.L.); wangshuying2021@sibs.ac.cn (S.W.)

**Keywords:** FGF21, mouse model, dietary pattern, low-protein diet, fasting

## Abstract

**Background**: Fibroblast growth factor 21 (FGF21), a secreted protein, plays a crucial role in regulating metabolism and energy homeostasis. Nevertheless, the expression pattern of Fgf21 across diverse tissues and its responsiveness to various dietary regimens remain incompletely understood. **Methods**: In this study, we developed a Fgf21-enhanced green fluorescent protein (EGFP) reporter mouse model to explore the expression of endogenous Fgf21 in different tissues under four dietary conditions: normal chow, low-protein diet, fasting, and fasting-refeeding. **Results**: A low-protein diet was found to induce Fgf21 expression in both the liver and skeletal muscle. Notably, Fgf21 was predominantly expressed in the periportal region of the liver. In the pancreas, Fgf21 exhibited a patchy expression pattern in the exocrine portion, but was absent in the endocrine part, regardless of the dietary regimens. Regarding the spleen, fasting triggered the expression of Fgf21, which was mainly localized in the red pulp area. Moreover, under fasting conditions, Fgf21 showed a scattered expression pattern in the small intestine. **Conclusions**: The Fgf21-EGFP reporter mouse model serves as a valuable tool for dissecting the expression of endogenous Fgf21 in different tissues under various dietary and stress conditions. Further investigations using this model may contribute to uncovering the hitherto unrecognized functions of locally produced FGF21.

## 1. Introduction

Fibroblast growth factor 21 (FGF21) is a member of the fibroblast growth factor (FGF) family. Ever since its discovery in 2000 [1], FGF21 has been acknowledged as a stress-induced hormone that plays a crucial role in mediating metabolic adaptation. This adaptation occurs in response to various stressors, such as nutrient starvation, cold exposure, alcohol consumption, and drug overdose [2,3,4,5]. FGF21 exerts its regulatory functions by interacting with a receptor complex made up of FGF receptors (FGFR1c and FGFR3c) and β-klotho. Notably, β-klotho determines the tissue-specific actions of FGF21 [6]. A wide array of metabolic processes have been found to be regulated by FGF21. Initially, FGF21 was identified for its ability to enhance glucose uptake in adipocytes [2]. Subsequently, it was discovered to be a potent insulin sensitizer, primarily through its direct action on adipose tissues [4,7]. Moreover, FGF21 promotes weight loss by increasing energy expenditure via a direct impact on the central nervous system [8]. Recent investigations involving FGF21 analogs/mimetics have clearly illustrated their beneficial effects on lipid metabolism. These effects include improvements in dyslipidemia, alleviation of hepatic steatosis, and mitigation of metabolic dysfunction-associated steatohepatitis (MASH) [5]. Presently, numerous FGF21 analogs/mimetics are being tested in clinical trials for the treatment of metabolic disorders, with a particular focus on liver diseases [9].

The basal level of FGF21 in many tissues, including the liver, is low, and its expression is stimulated by nutritional and stress signals. Studies using different dietary combinations have revealed that Fgf21 expression is mainly induced by low-protein intake combined with high-carbohydrate intake [10]. Nutritional deprivation, such as extended fasting, rapidly induces Fgf21 expression in the liver through the transcription factor peroxisome proliferator-activated receptor α (PPARα) [11,12]. Hepatic Fgf21 is induced by high-sugar intake, which depends on the transcription factor carbohydrate response element binding protein (ChREBP) [13]. On the other hand, protein restriction induces hepatic Fgf21 expression through pathways dependent on the transcription factors activating transcription factor 4 (ATF4) and nuclear respiratory factor (NRF) [14]. In humans, Fgf21 expression can be induced by starvation [15,16], protein restriction [17], high-fructose or carbohydrate intake [18,19], and alcohol consumption [20,21]. Intriguingly, FGF21 can suppress sweet and alcohol preferences through its central actions [22], further suggesting that FGF21 is a hormone involved in the adaptive response to food intake.

The circulating level of FGF21 during nutrient deprivation and stress mainly originates from the liver [23], highlighting the liver’s importance as the primary organ for stress-induced FGF21 expression. Fgf21 mRNA can be detected in many other tissues, such as the pancreas, skeletal muscle, and adipose tissue [24]. Fgf21 is expressed in the exocrine pancreas and regulates the secretion of digestive enzymes to maintain acinar cell proteostasis [25]. In pancreatitis, the expression of Fgf21 in pancreatic cells is significantly increased, and the severity of injury is inversely related to the expression of Fgf21 [26]. FGF21 in adipose tissues is thought to have an autocrine or paracrine function in regulating glucose uptake, beiging, and thermogenesis [27,28]. FGF21 has also been identified as a myokine in response to the inhibition of the mitochondrial function in skeletal muscle. It interacts with adipose tissue to regulate its browning [29], and autophagy deficiency in skeletal muscle protects mice from obesity and insulin resistance by stimulating the production of this myokine [30]. Later, it was discovered that FGF21 in skeletal muscle responds to impaired mitochondrial fatty acid oxidation and has a paracrine action in regulating glucose uptake [31].

In this study, we aimed to establish an Fgf21 reporter mouse model to help clarify the in vivo expression pattern of this hormone under different dietary conditions. Previously, a bicistronic reporter mouse using luciferase was reported to monitor Fgf21 expression [32]. It was found that fasting can induce a modest upregulation of luciferase activity in the liver [32]. However, the luciferase-based reporter analysis cannot clarify the cellular distribution of FGF21. Here, we placed a green fluorescent protein (GFP) reporter immediately following the start codon of the Fgf21 gene. We analyzed the GFP reporter under various dietary patterns. As expected, we found that a low-protein diet strongly elevated GFP fluorescence in the liver. Furthermore, we found that the in vivo expression of Fgf21 is detected in many other tissues, with different responses to various dietary regimens.

## 2. Materials and Methods

**Figure 1:**
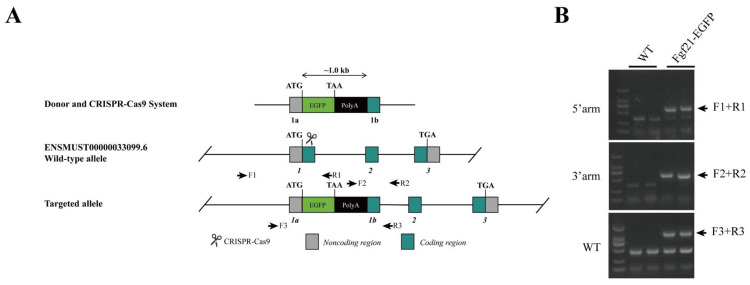
Construction of the Fgf21-EGFP reporter mouse. (**A**) The vector was constructed using the crisper-cas9 technology and then microinjected into the fertilized eggs of mice with a C57BL/6JGpt background, and the F0 generation of the recipient mice was genotyped and mated with wild-type background mice to select Fgf21-EGFP heterozygous mice for experiments. The primers used in genomic identification are indicated. (**B**) Results of genomic PCR using the primers as indicated.

Mice: The Fgf21-EGFP mice were procured from GemPharmatech Co., Ltd. (Nanjing, China). In this study, only male mice on the C57BL/6 genetic background were utilized. We chose the founders based on whether or not GFP signals were apparent in the liver after the mice were fed with a low-protein diet for 3 days. As a result, we obtained four mouse founders that were well responsive to the low-protein diet (#7, #12, #13, and #18). We used offsprings of founder #7 for all the experiments in this study. We used heterozygous mice throughout this study. For the protein restriction experiment, 8-week-old mice were fed a low-protein diet (LPD) for 3 days. The LPD, sourced from Jiangsu Synergistic Biological Co., had a composition of 5% protein, 71% carbohydrate, and 10% fat. In the fasting and refeeding experiments, the mice were fasted for 24 h and then refed with normal chow for 2 h. The composition of the diets used in this study is given in Table S1. The mice were housed in a specific-pathogen-free (SPF) facility with a 12 h light/12 h dark cycle and had free access to food and water. All animal experimental protocols were approved by the Institutional Animal Care and Use Committee of the Shanghai Institute of Nutrition and Health, Chinese Academy of Sciences, with an approval number SINH-2024-CY-1. Genotyping of the mice was carried out using the PCR method. Genomic DNA was isolated from the mouse tails. The primers employed for mouse identification were as follows: 5′-GGTATTTCTGCGTTCACCAGACAG-3′ and 5′-GCTTCAGTGTCTTGGTCGTCATC-3′ for wild type, 5′-GGTATTTCTGCGTTCACCAGACAG-3′ and 5′-GCTGTTGTAGTTGTACTCCAGCTTG-3′ for the 5′ arm, and 5′-ACAACCACTACCTGAGCACCCAGT-3′ and 5′-GCTTCAGTGTCTTGGTCGTCATC-3′ for the 3′ arm (Figure 1B).

Frozen sections: Fresh tissues, including the liver, pancreas, small intestine, and spleen, were excised and fixed in paraformaldehyde for 4 h. Subsequently, they were washed three times in a PBS solution for 30 min each. After that, the tissues were placed successively into a 15% sucrose solution and a 30% sucrose solution for overnight dehydration. Following dehydration, the tissues were washed three times in PBS for 5 min each, blotted dry, embedded in optimal cutting temperature compound (OCT) gel, and stored in a −80 °C refrigerator. Fresh skeletal muscle tissues were removed and directly embedded in OCT gel. They were then rapidly frozen in thawed isopentane for approximately 1 min, transferred to liquid nitrogen, and stored at −80 °C. Tissue sections (4 μm thick) were cut at −20 °C using a cryostat (Leica, Wetzlar, Germany), collected onto SuperFrost Plus (Thermo Fisher Scientific, Waltham, MA, USA) adhesive slides, and stored at −80 °C.

Immunofluorescence: Frozen sections were equilibrate at room temperature for 15 min and were then rehydrated in phosphate-buffered saline (PBS) for an additional 15 min. Nuclear staining was carried out using Hoechst 33342 (H3570, Invitrogen, Waltham, MA, USA). Specific antibodies were used to determine co-localization with insulin (3014S, CST), epithelial cadherin (14472T, CST), or glutamine synthetase (11037-2-AP, Proteintech, Rosemont, IL, USA). The sections were blocked and permeabilized in a PBS blocking buffer containing 5% goat serum and 0.4% Triton X-100 for 1 h. The primary antibodies were diluted in the blocking buffer and incubated overnight at 4 °C. Subsequently, the secondary antibody, also diluted in the blocking buffer, was incubated for 1 h at room temperature. After the incubation with the primary antibody, the sections were washed three times for 5 min each in PBS. Then, they were incubated with goat anti-rabbit IgG (H + L) Highly Cross-Adsorbed Secondary Antibody, Alexa Fluor™ 546 (A-11035, Thermo Fisher Scientific). Following this, the sections were washed three times for 5 min each in PBS and fixed with Fluoromount (F4680, Sigma-Aldrich, St. Louis, MO, USA). The slides were imaged using a Zeiss LSM880NLO FLIM confocal microscope (Carl Zeiss, Jena, Germany).

Reverse transcript quantitative PCR (RT–qPCR): Total RNA of the tissues was isolated using the guanidine isothiocyanate-phenol-chloroform extraction method. The mRNA expression was quantified using the QuantStudio 6 Flex with QuantStudio Real-Time Systems LightCycler (Thermo Fisher Scientific) with SYBR Green. The primers for detecting Fgf21 expression were 5′-CTGGGGGTCTACCAAGCATA-3′ and 5′-CACCCAGGATTTGAATGACC-3′.

Statistical Analysis: Unpaired two-tailed Student’s *t*-test was performed to determine the statistical significance of the differences. *p* values equal to or less than 0.05 were considered statistically significant.

## 3. Results and Discussion

We engineered the Fgf21-enhanced green fluorescent protein (EGFP) reporter mice by inserting the EGFP gene immediately downstream of the start codon of the Fgf21 gene (Figure 1A). To generate these reporter mice, gRNA was first designed, constructed, and transcribed in vitro. Concurrently, a homologous recombinant vector (donor vector) was constructed, and its sequence was verified through sequencing (Figure 1A). Subsequently, samples of the CRISPR/Cas9 system and the donor vector were microinjected into fertilized eggs of C57BL/6JGpt-background mice. The surviving fertilized eggs were then transplanted into pseudo-pregnant female mice, which carried the embryos to term and gave birth to pups. The F0 littermates born to the recipient mice were numbered, and at 5–7 days old, samples of their tails and toes were collected. Genomic DNA was extracted from these samples and subjected to PCR and sequencing for genotype confirmation. Once the positive F0-generation mice reached sexual maturity, they were mated with wild-type background mice. The F1-generation mice were born, and at 5–7 days old, their tails and toes were sampled and numbered. Genomic DNA was extracted, and genomic PCR was performed to confirm the Fgf21-EGFP genotype (Figure 1B).

**Figure 2:**
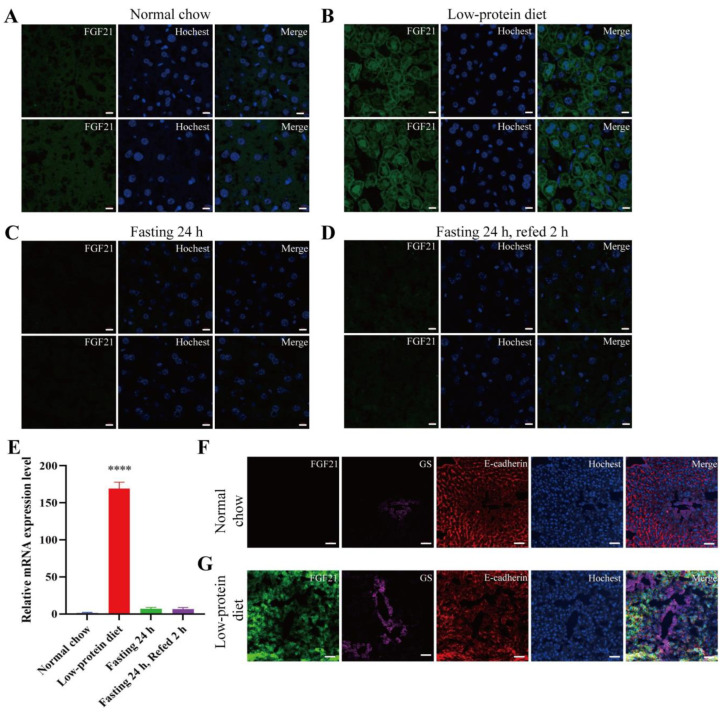
Low-protein diet stimulates Fgf21 expression in the liver. (**A**–**D**) Representative images of GFP signals detected by immunofluorescence in liver sections. Mouse samples were isolated from 8-week-old male mice treated with different dietary regimens (n = 3 mice for each group). Fresh tissues were fixed, dehydrated and embedded immediately after the dietary cycle. Frozen sections were prepared and stained. The nuclei were stained with Hoechst 33342. Scale bar: 10 μm. (**E**) The mRNA levels of the Fgf21 gene in liver as detected by quantitative RT-PCR (n = 3 mice for each group). Data are shown as mean ± SEM. **** *p* < 0.0001 as compared to the first group. (**F**,**G**) Immunofluorescence staining for glutamine synthetase (GS) and E-cadherin with the liver sections. Scale bar: 50 μm.

In this study, we utilized the Fgf21-EGFP reporter mice to analyze the in vivo expression pattern of Fgf21 under four distinct dietary regimens: a normal chow diet, a 3-day low-protein diet (LPD), 24 h fasting, and 24 h fasting followed by 2 h refeeding. Under a normal diet, the expression level of FGF21 in the liver was relatively low (Figure 2A). In contrast, the LPD strongly induced Fgf21 expression in the liver, presenting a distinct grid-like green fluorescence pattern (Figure 2B). Fasting and fasting-refeeding did not visibly stimulate Fgf21 expression in the liver (Figure 2C,D). RT-PCR was conducted to confirm Fgf21 expression, and the results showed that only the LPD significantly induced Fgf21 expression in the liver (Figure 2E). Given that our low-protein diet consisted of 5% protein and 71% carbohydrate, our findings align with the concept that hepatic Fgf21 is mainly induced by a combination of low protein and high carbohydrate [10].

Anatomically, the central venous area (CV) of the liver is located at the center of hepatic lobules, where it collects blood from hepatic sinusoids. The liver’s blood converges into the hepatic vein and ultimately returns to the heart. Conversely, the portal vein region (PV) is positioned at the periphery of hepatic lobules and transports nutrient- and oxygen-rich blood from the digestive tract to the liver. Glutamine synthetase (GS) serves as a marker for pericentral hepatocytes, while epithelial cadherin (E-cadherin) is a marker for periportal hepatocytes. We conducted a further investigation into the localization of GFP-positive cells under a low-protein diet. Immunofluorescent co-localization analysis demonstrated that the GFP-positive cells did not co-localize with GS but did co-localize with E-cadherin (Figure 2F,G). This suggests that, under a low-protein diet, Fgf21 is mainly expressed in the periportal region of the liver. Intriguingly, our result aligns with a previous report indicating that Fgf21 is expressed in the periportal region of the mouse liver during leucine deprivation [33].

**Figure 3:**
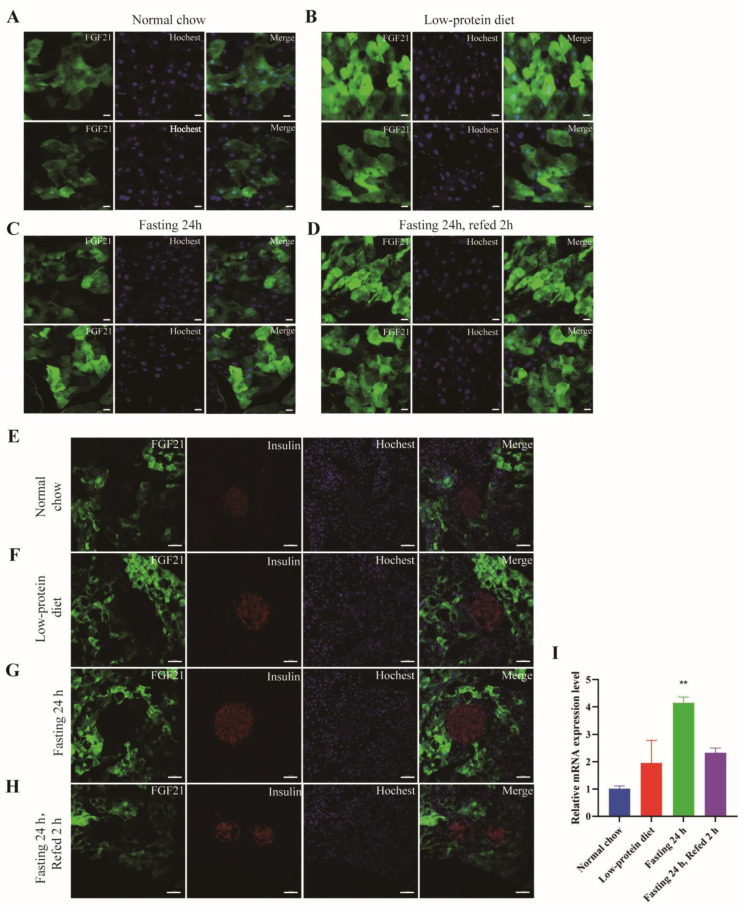
FGF21 is abundantly expressed in exocrine pancreas. (**A**–**D**) Representative images of the GFP signals detected by immunofluorescence in pancreas sections from the mice as in Figure 2. The nuclei were stained with Hoechst 33342. Scale bar: 10 μm. (**E**–**H**) Immunofluorescent staining for insulin and Hochest 33342 in the pancreas. Scale bar: 50 μm. (**I**) The mRNA levels of Fgf21 as detected by quantitative RT-PCR (n = 3 mice for each group). Data are shown as mean ± SEM. ** *p* < 0.01, as compared to the first group.

By using the Fgf21-EGFP reporter mice, we observed consistent GFP signaling in the exocrine pancreas, regardless of the dietary patterns (Figure 3A–D). In addition, RT-PCR result demonstrated that a low-protein diet could significantly increase the Fgf21 mRNA level in the pancreas (Figure 3I), suggesting that Fgf21 expression in the pancreas is elevated by fasting. Notably, the GFP signals in the exocrine pancreas exhibited a distinct patchy distribution pattern. In some parts of the exocrine pancreas, there was no EGFP signal at all. This observation further validates the finding that FGF21 functions as a secretagogue in the exocrine pancreas [25]. Based on our observations, we propose that the exocrine pancreas can be divided into two types of cells: Fgf21-positive and Fgf21-negative exocrine cells. Elucidating whether these two cell types play distinct roles in the exocrine function of the pancreas will be of significant importance. In addition, we stained pancreatic islets with an insulin-specific antibody. We found that under the four dietary conditions, the GFP signal was absent in pancreatic islets (Figure 3E–H). This suggests that Fgf21 is mainly expressed in the exocrine part rather than the endocrine part of the pancreas.

**Figure 4:**
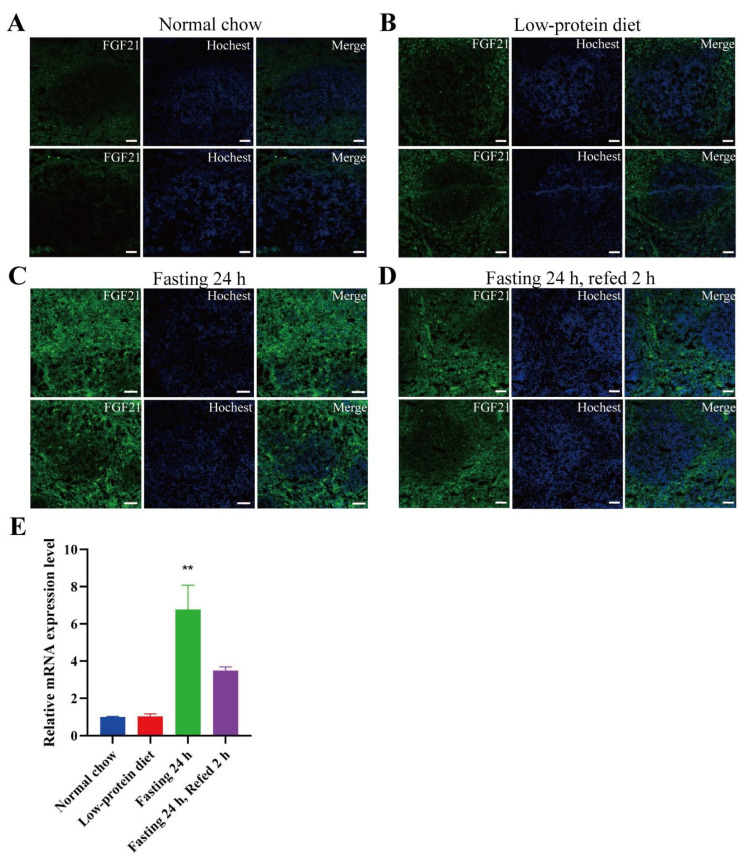
Fasting stimulates Fgf21 expression in the spleen. (**A**–**D**) Representative images of GFP signals detected by immunofluorescence in the spleen sections from the mice as in Figure 2. The nuclei were stained with Hoechst 33342. Scale bar: 50 μm. (**E**) The mRNA level of Fgf21 in the spleen (n = 3 mice for each group). Data are shown as mean ± SEM. ** *p* < 0.01 as compared to the first group.

Another organ in which detectable GFP signals were present was the spleen. The spleen is composed of both white and red pulp. The white pulp, mainly consisting of lymphoid tissue densely populated with lymphocytes, manifests as scattered grayish-white dots in fresh spleen sections. The red pulp contains a large number of hemocytes and macrophages. Under a normal chow diet, the GFP signals in the spleen were extremely weak (Figure 4A). They were slightly enhanced under a low-protein diet (Figure 4B) and became highly evident under fasting and fasting-refeeding conditions (Figure 4C,D). RT-PCR using the spleen tissue also revealed that fasting could significantly elevate the Fgf21 mRNA level (Figure 4E). These data imply that fasting can effectively stimulate Fgf21 expression in the spleen. Additionally, we observed that the GFP signals were predominantly localized in the red pulp area (Figure 4). To the best of our knowledge, the expression of Fgf21 in the spleen has not been previously reported. Since the red pulp of the spleen is a key site for macrophage phagocytosis and antigen presentation to lymphocytes, we hypothesize that the fasting-induced Fgf21 in the red pulp region may exert an autocrine or paracrine function within this area of the spleen.

**Figure 5:**
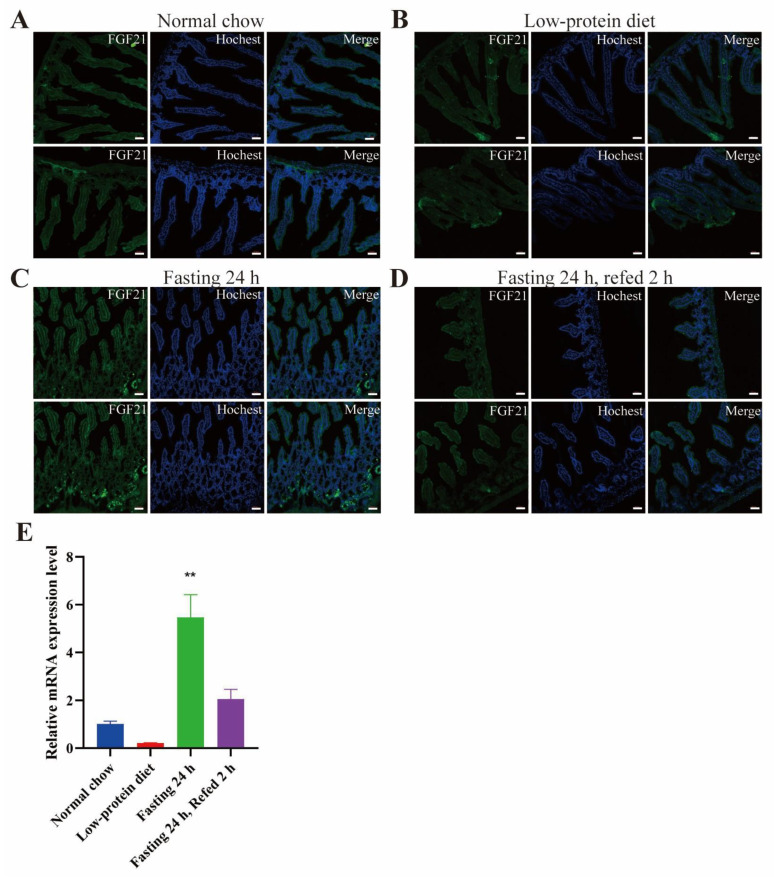
Fasting increases Ffg21 expression in the small intestine. (**A**–**D**) Representative images of GFP signals detected by immunofluorescence in sections of small intestine from the mice as in Figure 2. The nuclei were stained with Hoechst 33342. Scale bar: 50 μm. (**E**) The mRNA level of Fgf21 in the small intestine (n = 3 mice for each group). Data are shown as mean ± SEM. ** *p* < 0001 as compared to the first group.

We also detected a scattered distribution of GFP signals in the small intestine, mainly under fasting conditions (Figure 5). The low-protein diet and fasting-refeeding seemed to decrease the GFP signals in the small intestine (Figure 5B,D). Analysis of RT-PCR also showed that the Fgf21 mRNA level was increased by fasting (Figure 5E). The expression of endogenous Fgf21 in the small intestine has not been reported hitherto. However, it has been found that an FGF21 treatment can lower glucose levels in diabetic mice, partly by inhibiting glucose absorption in the small intestine [34]. Thus, it would be fascinating to explore whether the locally produced FGF21 has an autocrine/paracrine function in regulating glucose uptake in the small intestine.

**Figure 6:**
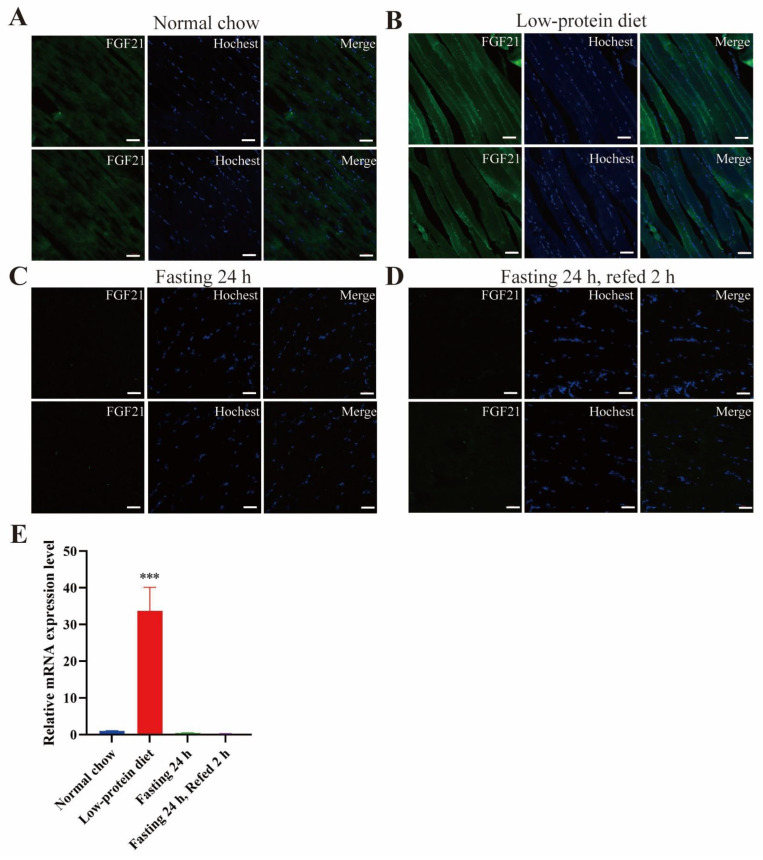
Low-protein diet stimulates Fgf21 in skeletal muscle. (**A**–**D**) Representative images of GFP signals detected by immunofluorescence in sections of skeletal muscle (quadriceps) from the mice as in Figure 2. The nuclei were stained with Hoechst 33342. Scale bar: 50 μm. (**E**) The mRNA levels of FGF21 in the skeletal muscle (n = 3 mice for each group). Data are shown as mean ± SEM. *** *p* < 0.001 as compared to the first group.

In the Fgf21-EGFP reporter mice, distinct GFP signals were observed in the skeletal muscle (Figure 6). Analogous to the findings in the liver, the GFP signals in the skeletal muscle were significantly enhanced by a low-protein diet (Figure 6B). After 24 h of fasting, the GFP intensity was markedly reduced, and refeeding did not alter this intensity (Figure 6C,D). Consistently, RT-PCR results demonstrated that the low-protein diet significantly elevated the mRNA level of Fgf21 in the skeletal muscle (Figure 6E). These observations were further validated by RT-PCR results (Figure 6E). Evidently, these findings clearly demonstrate that endogenous Fgf21 expression in the skeletal muscle is induced by protein restriction. Given that FGF21 has been proposed to function as a myokine, interacting with adipose tissue to regulate browning [29], the FGF21 derived from skeletal muscle under a low-protein diet may partially account for the beneficial effects of protein restriction on metabolic health and aging [35,36].

It is important to note that the mouse FGF21 protein has a signal peptide of 29 amino acid residues at the N-terminus. This signal peptide is responsible for guiding the secretion of the FGF21 protein out of the cells. In our Fgf21-EGFP reporter mouse model, a GFP reporter was inserted immediately after the start codon of the Fgf21 gene. As a result, the signal peptide is absent. Without the signal peptide, the EGFP cannot be secreted and is instead expressed nonspecifically within the cytoplasm. Consequently, this reporter mouse model is unsuitable for analyzing the subcellular localization of FGF21. Nevertheless, these reporter mice can serve as a valuable tool for clarifying the tissue expression pattern of FGF21 under various nutritional and pathophysiological conditions.

## 4. Conclusions

In summary, by utilizing the Fgf21-EGFP reporter mouse model, we discovered that the in vivo expression of Fgf21 in different tissues/organs responds diversely to various dietary patterns. A low-protein diet predominantly stimulates Fgf21 expression in the liver and skeletal muscle. Fgf21 exhibits a patchy expression pattern in the exocrine pancreas, while being absent in the endocrine pancreas, regardless of the dietary regimens. In the spleen, Fgf21 is mainly expressed in the red pulp area in response to fasting. Fgf21 also shows scattered expression in the small intestine, primarily under fasting conditions. Thus, the Fgf21-EGFP reporter mouse model proves to be a valuable tool for dissecting the expression of endogenous Fgf21 in different tissues/organs under diverse diets and stress conditions. Further exploration of this reporter model may assist in uncovering the previously unrecognized functions of locally produced FGF21.

## Data Availability

The data presented in this study are available on request from the corresponding author. The data are not publicly available due to privacy reasons.

## Supplementary Materials

The following supporting information can be downloaded at: https://www.mdpi.com/article/10.3390/nu17071179/s1, Table S1: Composition of the mouse diet.

## Abbreviations

The following abbreviations are used in this manuscript:

ATF4	activating transcription factor 4
ChREBP	carbohydrate response element binding protein
EGFP	enhanced fluorescence protein
FGF21	Fibroblast growth factor 21
GFP	green fluorescence protein
LPD	low-protein diet
MASH	metabolic dysfunction-associated steatohepatitis
NRF	nuclear respiratory factor
OCT	optimal cutting temperature compound
PPARα	peroxisome proliferator-activated receptor α
