# Supplementary material for: Distinct Fgf21 Expression Patterns in Various Tissues in Response to Different Dietary Regimens Using a Reporter Mouse Model
Source: Nutrients. 2025 Mar 28;17(7):1179. doi: 10.3390/nu17071179 (PMC11990235; doi:10.3390/nu17071179)
Supplement: Supplementary file 1 [file nutrients-17-01179-s001.zip › nutrients-3539392-supplementary.pdf]

Supplemental Table 1. Composition of the mouse diet

	AIN-93G (normal chow)		Low-protein diet	
	gm%	kcal%	gm%	kcal%
Protein	20.30%	20.30%	5.12%	5.12%
Carbohydrate	63.95%	63.95%	79.13%	79.13%
Fat	7.00%	15.75%	7.00%	15.75%
**Total**		**100.00%**		**100.00%**
**Ingredient**	**gm**	**kcal**	**gm**	**kcal**
Casein	200	800	50	200
L-Cystine	3	12	1.2	4.8
Corn Starch	397.5	1590	549.3	2197.2
Maltodextrin 10	132	528	132	528
Sucrose	100	400	100	400
Cellulose	50	0	50	0
Soybean Oil	70	630	70	630
Vitamin Mix V10037	10	40	10	40
Mineral Mix S10022G	35	0	35	0
Choline Bitartrate	2.5	0	2.5	0
**Total**	**1000**	**4000**	**1000**	**4000**
